# Parallel divergent adaptation along replicated altitudinal gradients in Alpine trout

**DOI:** 10.1186/1471-2148-12-210

**Published:** 2012-10-27

**Authors:** Irene Keller, Jolanda Schuler, Etienne Bezault, Ole Seehausen

**Affiliations:** 1 Department of Fish Ecology and Evolution, EAWAG Swiss Federal Institute of Aquatic Science and Technology, Center of Ecology, Evolution and Biogeochemistry, Seestrasse 79, 6047 Kastanienbaum, Switzerland; 2 Department of Aquatic Ecology, EAWAG Swiss Federal Institute of Aquatic Science and Technology, Überlandstrasse 133, 8600, Dübendorf, Switzerland; 3 Department of Aquatic Ecology and Macroevolution, Institute of Ecology and Evolution, University of Bern, Baltzerstrasse 6, 3012 Bern, Switzerland; 4 Present address: Department of Biology, Reed College, 3203 SE Woodstock Blvd, Portland, OR, 97202-8199, USA

**Keywords:** European trout, Local adaptation, Genome scan, AFLP, Environmental gradient, Parallel adaptation

## Abstract

**Background:**

The European trout (*Salmo trutta* species complex) occurs across a very wide altitudinal range from lowland rivers to alpine streams. Historically, the major European river systems contained different, evolutionarily distinct trout lineages, and some of this genetic diversity has persisted in spite of extensive human-mediated translocations. We used AFLP-based genome scans to investigate the extent of potentially adaptive divergence among major drainages and along altitudinal gradients replicated in several rivers.

**Results:**

The proportion of loci showing evidence of divergent selection was larger between drainages than along altitudinal transects within drainages. This suggests divergent selection is stronger between drainages, or adaptive divergence is constrained by gene flow among populations within drainages, although the latter could not be confirmed at a more local scale. Still, altitudinal divergence occurred and, at approximately 2% of the markers, parallel changes of the AFLP band frequencies with altitude were observed suggesting that altitude may well be an important source of divergent selection within rivers.

**Conclusions:**

Our results indicate that adaptive genetic divergence is common both between major European river systems and along altitudinal gradients within drainages. Alpine trout appear to be a promising model system to investigate the relative roles of divergent selection and gene flow in promoting or preventing adaptation to climate gradients.

## Background

The environment experienced by particular populations often varies across the distribution range of a species, producing spatial heterogeneity in the direction and strength of natural selection. Such divergent selection may lead to the evolution of traits beneficial under the environmental conditions specific to a particular population, a process known as local adaptation [1,2]. When adaptation occurs to a recurrent, well-defined habitat type (e.g. highland versus lowland), distinct ecotypes may form which are usually characterized by divergence in several traits [3]. The extent of adaptive differentiation between populations and ecotypes will often reflect a balance between differentiating effects of selection and homogenising effects of gene flow [4,5]. The relative importance of these two factors may be particularly amenable to study along environmental gradients where habitat changes occur at a small spatial scale relative to the dispersal distance of an organism.

Theory shows that local adaptation can evolve along such gradients in the face of gene flow [6-8] and can set the stage for ecotype formation and ecological speciation [9]. Many empirical examples exist, where the frequency of a particular trait or allele changes along the gradient in a clinal manner (see e.g. [10,11]), and examples exist too for ecotype formation along environmental gradients [12,13]. The ecological changes along environmental gradients may be very complex involving covariation between multiple abiotic and biotic variables. Along altitudinal gradients, for example, we expect a pronounced decrease in ambient temperature with increasing altitude but concomitant patterns may include a reduction in the diversity and a change in the composition of local communities of prey, competitors, parasites and pathogens [14-16]. These changes are expected to result in divergent selection on a range of traits, and genetic changes correlated with altitude have been reported from various species [17,18], including salmonid fishes [19,20].

**Figure 1:**
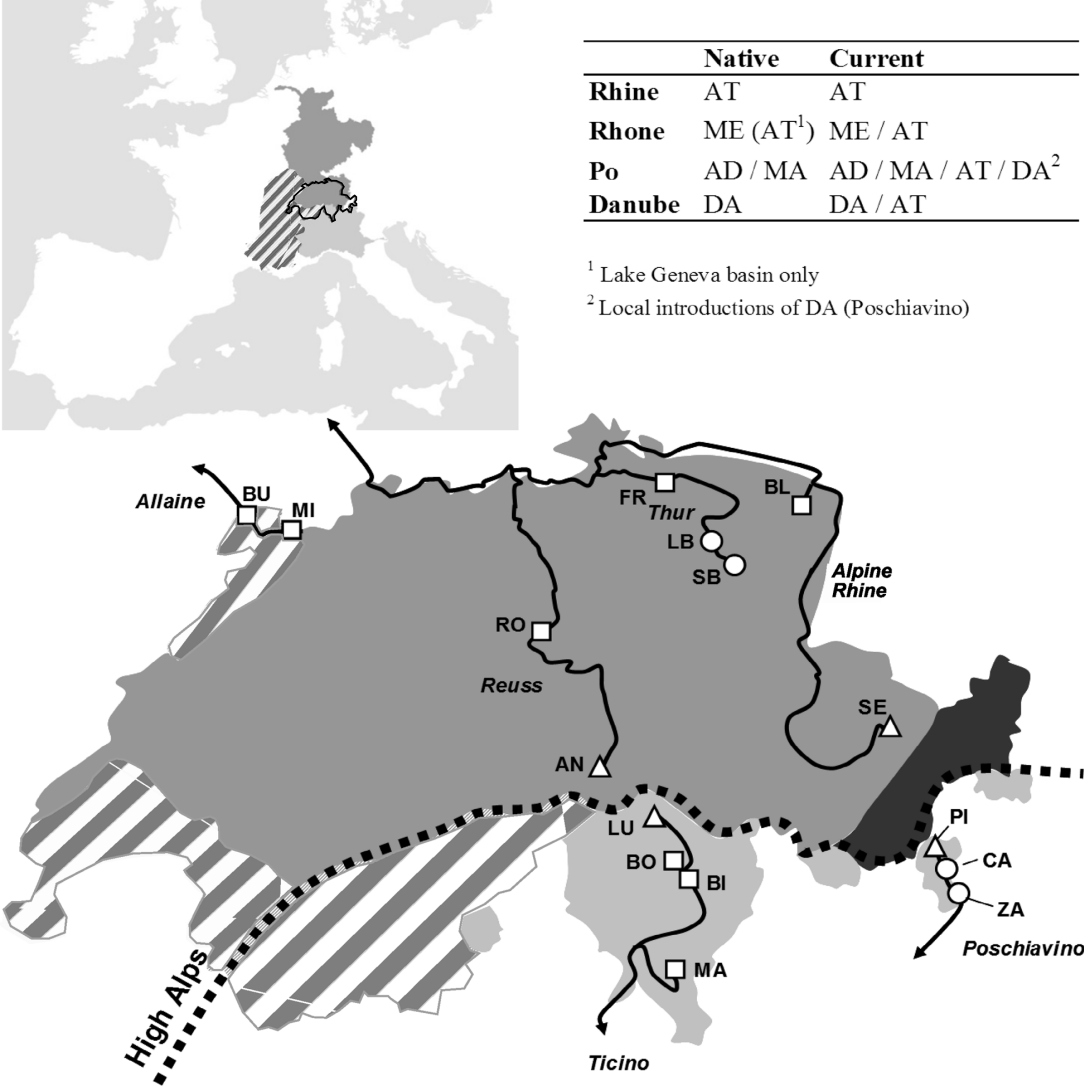
**Sampling sites along altitudinal transects replicated in the Alpine section of three major European drainages. **Major drainages are indicated by different shading (medium grey=Rhine; light grey=Po; hatched=Rhone; dark grey=Danube) and river systems within drainages are labelled in italics. Different symbols indicate different altitudinal zones: squares < 500m, circles = 500-1000m, triangles >1000m above sea level. Inset table: Native and current distribution of five major evolutionary trout lineages (based on Largiadèr & Scholl 1995, 1996) corresponding to different haplogroups of Bernatchez (2001) and species (in parentheses) of Kottelat & Freyhof (2007): AT=Atlantic (*S. trutta*), ME=Mediterranean (*S. rhodanensis*), AD=Adriatic (*S. cenerinus*), MA=marble trout (*S. marmorata*), DA=Danubian (*S. labrax*).

The European trout (*Salmo trutta* species complex) lives in an environment that is heterogeneous at several different spatial scales. First of all, consistent environmental differences may exist between major European river basins, and trout populations may have evolved drainage-specific adaptations during extended periods of allopatric divergence. In fact, the major European drainages historically contained distinct trout lineages which probably split in the Pleistocene some 0.5 to 2 million years ago ([21]; see Figure 1) and are mostly considered separate species in a recent taxonomic reassessment of European trouts [22]. The extent and nature of adaptive divergence between these lineages are poorly understood but lineage-specific differences in coding sequences [23,24] may be the result of divergent selection between drainages. At a more local scale, the environment varies across the wide altitudinal range occupied by trout, with similar gradients replicated in each drainage.

During most of the evolutionary history of trout, the rates of gene flow will have been much higher within than among drainages, and this is particularly true in the Alps. However, in the recent past (mostly the last century; [25]), the isolating effects of spatial distance and physical barriers have been partly offset by human management practices. In Switzerland, for example, large numbers of fish of Atlantic origin (*S. trutta*), which were historically restricted to the Rhine system and Lake Geneva basin, were introduced into the Rhone and Po until a change in fisheries legislation in 1991 banned such translocations. Several molecular studies showed that these introduced fish hybridised extensively with the native lineages and genetic variants historically associated with *S. trutta* now occur in all Swiss drainages [26-29]. Genetic variants typical of non-Atlantic lineages, on the other hand, remain strongly associated with particular drainages (*S. rhodanensis* (Mediterranean of Bernatchez 2001) in the Rhone; *S. cenerinus* (Adriatic) and *S. marmorata* (marble) in the Po; and *S. labrax* (Danubian) in the Danube; see Figure 1). Based on an analysis of putatively neutral and candidate microsatellite markers, we found in a previous study that the proportion of *S. trutta*-like alleles and genotypes varied between regions within the Mediterranean drainages [29]. Within the Po system, this *S. trutta* contribution was considerably higher in one studied area (Ticino) than another (Poschiavino). In a river from the Rhone drainage, we detected evidence for the coexistence of two distinct trout species, one corresponding to introduced *S. trutta* and the other most likely representing the native *S. rhodanensis*.

Here, we extend the genomic coverage for a subset of the samples from Keller et al. (2011) with 229 amplified fragment length polymorphism (AFLP) markers to investigate more specific hypotheses with respect to the role of phylogeographic history, environmental contrast and current gene flow on putatively adaptive population divergence. In particular, the higher genomic coverage substantially increases our power to detect patterns of parallel genetic divergence within rivers and provides an estimate of the proportion of loci potentially under divergent selection. A particular focus of the current analyses is on patterns of adaptive genetic divergence between major European drainage systems, a question that has not been addressed in detail before. Specifically, we ask:

1) Do we detect evidence of adaptive genetic divergence between trouts from different major European drainage systems? In the past, this would have amounted to a comparison between different trout species of Pleistocene origin. Today, it corresponds to a comparison between populations of pure *S. trutta* origin (Rhine) and trout with a potentially admixed genomic composition (Po: *S. cenerinus*/*S. marmorata* admixed with *S. trutta* and, locally, *S. labrax*; Rhone: *S. rhodanensis* admixed with *S. trutta*; see Figure 1). We expect loci involved in drainage-specific adaptation to have retained elevated levels of differentiation between drainages in the face of human-mediated gene flow. The coexistence of two partially isolated trout forms within the Rhone provides a unique opportunity to investigate if the same genetic variants are repeatedly favoured in the same environment but different genomic backgrounds.

2) Do we detect parallel associations between the frequency of particular genetic variants and altitude in different rivers? To this end, we compare patterns of genetic differentiation along altitudinal transects replicated in three major European drainage systems (Rhine, Rhone, Po). Parallel clines at the same genetic loci are expected if adaptation involves either genetic variants predating the split between the trout species, parallel mutation, or genetic variants historically associated with *S. trutta*, which were spread outside the Rhine system through stocking and subsequently recruited for altitudinal adaptation.

3) How does i) the environmental contrast and ii) the potential for natural dispersal between sites influence the extent of adaptive divergence? Specifically, we predict a positive correlation for the former if larger environmental differences lead to stronger divergent selection and a negative correlation for the latter if adaptive divergence is constrained by gene flow. To address these questions, we use the proportion of loci showing significantly elevated levels of between-population differentiation (i.e. outlier behaviour in a genome scan; e.g. [30,31]) as a measure of putatively adaptive divergence (see e.g. [32]).

## Results

We sampled trout from 16 sites along altitudinal gradients replicated within and between three major European drainage systems, Rhine, Rhone and Po (Figure 1). Using 12 primer pairs, we obtained amplified fragment length polymorphism (AFLP) data for 229 polymorphic loci in 369 individuals with a genotype reproducibility >95.3%.

### Remnants of phylogeographic structure in spite of human-mediated dispersal

**Figure 2:**
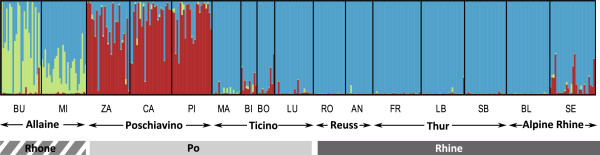
**Results of Structure analysis assuming K=3 based on 229 AFLP markers and samples from 16 locations in three major European drainages. **Population labels correspond to Figure 1. The different drainages are indicated by the bar below the plot and the shading is consistent with Figure 1.

A Bayesian clustering algorithm implemented in the software Structure was used to investigate the genetic substructure in our dataset. We found that the support for a given number of clusters increased steeply up to K=3, with a slight further increase of the likelihood up to K=5. The geographical patterns were best resolved at K=3, where the genetic subdivisions corresponded well with the major drainage systems. The Rhine individuals, with the exception of population SE, were fully assigned to a single genetic cluster (blue in Figure 2), which was also observed at variable frequencies in all other drainages. A second cluster appeared in the Rhone (green in Figure 2) and a third in the Po (red in Figure 2), at considerably higher frequency in the Poschiavino than in the Ticino. In the Rhone (particularly in site BU), the distribution of the inferred ancestry proportions appeared bimodal: Some individuals were assigned mostly to the local cluster, others mostly to the cluster dominant in the Rhine with only few intermediates, suggesting the coexistence of two distinct trout species, one corresponding to introduced *S. trutta* and the other most likely representing the native *S. rhodanensis* (see also [29]). One Rhine population (SE) showed evidence of genetic admixture between the Rhine-like and the Po-like clusters.

The results of an analysis of molecular variance supported the subdivisions indicated by Structure. When sites were grouped by major drainages, 8.5% of the variation was observed among drainages and 6.2% among populations within drainages. An additional split between the two geographical regions sampled in the Po drainage (Ticino, Poschiavino), increased the between-drainage component to 10.8%. Note that the Poschiavino and Ticino river systems are treated as separate drainages in all subsequent drainage-based analyses.

The number of polymorphic markers was highest in the Poschiavino, intermediate in the Rhone and lowest in the Ticino and Rhine (Table 1). This pattern is, at least to some extent, contrary to the number of analysed individuals which was highest in the Rhine and lowest in the Rhone.

**Table 1:** List of putatively adaptive loci detected within drainages or individual rivers in Alpine trout populations

	**Outlier scan**							**Genotype-altitude association**	
**Drainage**	**Rhi**				**Rho**	**T**	**P**	**all**	**Rhi**
**River within drainage**		***Reuss***	***Thur***	***A-Rhi***					
# polymorphic markers	148	120	129	143	169	152	209	229	148
# pops	7	2	3	2	2	3	3	16	7
# ind	175	36	83	55	53	63	78	369	175
Loc1	**2.92**	NA	**1.76**	NA	NA	NA	0.01	0.38	**0.00**
Loc2	**0.99**	NA	0.18	NA	NA	NA	0.01	0.32	0.39
Loc3	**0.96**	NA	0.33	NA	−0.08	NA	−0.03	0.60	0.15
Loc4	**0.73**	NA	0.08	0.13	0.01	0.00	NA	0.25	0.07
Loc5	**0.71**	0.02	0.09	0.02	−0.06	−0.03	0.05	0.14	0.07
Loc6	**0.65**	0.11	0.10	0.14	0.02	−0.02	−0.02	**0.04**	**0.01**
Loc7	**0.58**	−0.01	**0.67**	NA	0.00	−0.02	−0.03	0.60	**0.01**
Loc8	**0.55**	0.01	0.06	−0.01	0.06	0.06	−0.04	0.39	**0.00**
Loc9	**0.46**	−0.01	0.08	−0.01	0.03	−0.06	−0.01	0.41	0.80
Loc10	**0.44**	0.03	0.06	0.06	0.06	NA	−0.03	0.24	0.28
Loc11	**0.42**	0.43	0.00	0.04	−0.09	−0.01	0.02	**0.03**	0.27
Loc12	**0.41**	NA	0.17	NA	NA	NA	−0.03	0.56	**0.00**
Loc13	**0.33**	−0.01	0.02	0.03	0.03	0.06	−0.01	0.41	0.36
Loc14	0.18	0.00	**0.73**	−0.03	−0.04	0.02	−0.03	**0.00**	0.07
Loc15	−0.04	NA	**0.43**	0.01	0.00	NA	0.04	0.61	0.90
Loc16	0.06	−0.03	**0.38**	NA	NA	0.01	−0.03	0.10	0.07
Loc17	0.33	−0.02	−0.10	**0.77**	−0.04	0.11	−0.01	0.12	**0.00**
Loc18	−0.10	0.01	NA	0.04	−0.05	**0.71**	−0.01	0.41	**0.00**
Loc19	−0.11	−0.03	0.06	0.01	−0.07	−0.07	0.02	**0.00**	**0.01**
Loc20	−0.08	0.01	0.07	−0.03	−0.02	0.03	−0.01	**0.00**	0.51
Loc21	NA	NA	NA	NA	−0.03	−0.04	−0.02	**0.01**	NA
Loc22	−0.21	−0.05	0.05	−0.01	−0.01	−0.06	0.02	**0.02**	**0.04**
Loc23	−0.13	NA	−0.07	0.03	−0.05	NA	−0.03	**0.02**	0.10
Loc24	−0.10	−0.02	−0.03	−0.05	−0.02	0.11	−0.02	**0.02**	**0.01**
Loc25	−0.05	0.00	−0.10	0.04	−0.05	0.24	−0.04	**0.03**	0.87
Loc26	−0.16	NA	NA	NA	NA	NA	NA	**0.03**	**0.00**
Loc27	NA	NA	−0.01	NA	0.00	NA	−0.02	**0.03**	NA
Loc28	−0.13	0.00	0.04	−0.03	−0.02	0.10	−0.02	**0.03**	**0.01**
Loc29	0.15	0.01	0.03	−0.01	0.01	−0.05	−0.01	**0.03**	0.06
Loc30	0.05	NA	NA	−0.01	NA	−0.06	−0.03	**0.04**	**0.04**
Loc31	−0.05	−0.03	0.01	NA	0.03	NA	0.00	**0.04**	0.78
Loc32	−0.07	NA	NA	0.00	0.01	NA	0.00	**0.04**	0.08
Loc33	−0.12	NA	NA	0.18	NA	NA	0.00	0.06	**0.00**
Loc34	0.00	−0.02	0.06	0.08	−0.04	−0.03	−0.02	0.13	**0.00**
Loc35	−0.11	NA	−0.03	−0.06	0.02	NA	−0.04	0.12	**0.00**
Loc36	−0.03	0.03	−0.03	−0.01	−0.04	−0.10	−0.02	0.65	**0.00**

**a) Signature of selection** (**Outlier scan):** Logarithm of the posterior odds for AFLP markers detected as significant outliers in at least one drainage- or river-specific analysis. Bold underlined font indicates tests significant at a threshold consistent with a false discovery rate of 20%.

**b) Genotype-altitude association: All:** Results of a permutation test assessing the association between AFLP band frequency and altitude accounting for drainage structure. Listed are all markers with a one-sided P-value ≤0.05 (bold and underlined). **Rhi:** P-values from a logistic regression testing for an association between AFLP band frequency and altitude within the Rhine. Listed are all markers significant after Bonferroni correction (bold and underlined). Bold type only indicates P-values ≤0.05 but not significant after multiple test correction.

To investigate the possibility of repeated but weak patterns, all results are shown for the listed markers. *Rhi* Rhine, *Rho* Rhone, *T* Ticino, *P* Poschiavino, *A*-*Rhi* Alpine Rhine. *NA* marker monomorphic or containing too much missing data.

### Divergent selection between drainages

We tested for signatures of adaptive genetic divergence between trouts from different drainages with outlier scans in Bayescan. The two geographically (and genetically; see above) distinct regions from the Po were treated as separate groups as were the two coexisting trout species, *S. trutta* and *S. rhodanensis*, within the Rhone. We performed all possible pairwise outlier scans between these five groups, with populations within drainages pooled. The results from separate analyses based on microsatellites and AFLPs were combined.

**Figure 3:**
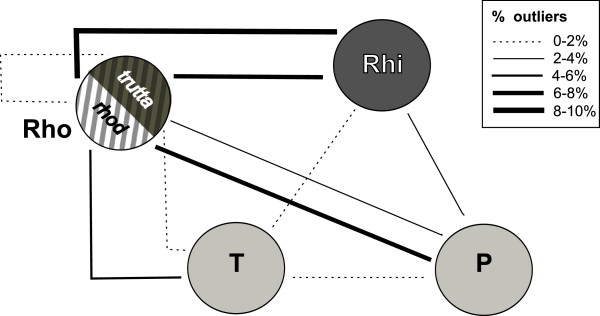
**Proportion of AFLP and microsatellite markers detected as outliers between drainages. **Within the Rhone, individuals were grouped as having a *S.trutta*-like (*trutta*) or a *S. rhodanensis*-like (*rhod*) genotype based on the results from a Structure analysis (see text for details), and separate outlier scans were conducted for these two genetic groups. Note that the shading of the circles is consistent with that used for the drainages in Figure 1. Rhi=Rhine, Rho=Rhone, T=Ticino, P=Poschiavino.

**Figure 4:**
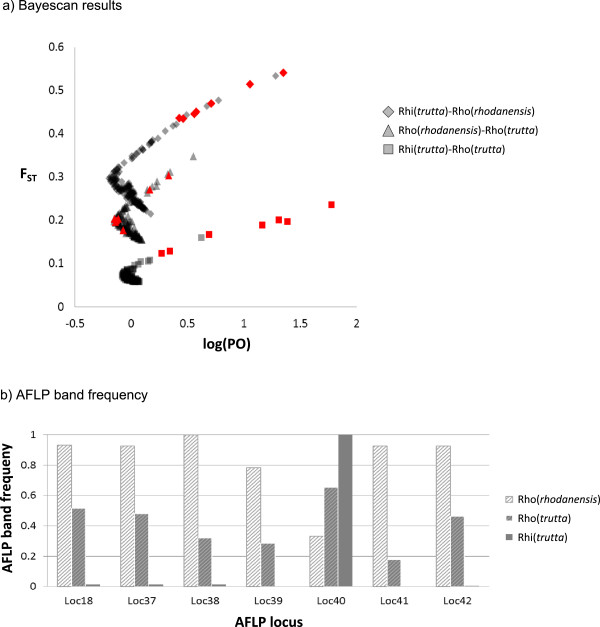
**Evidence for adaptive divergence between the Rhine and the Rhone and for adaptive introgression of *S. rhodanensis *variants into sympatric *S. trutta *in the Rhone. a**) Bayescan results: Logarithm of the posterior odds (PO) against F_ST_ for all AFLP markers in all three pairwise comparisons between *S. trutta* from the Rhine (Rhi) and the two species, *S. rhodanensis* and *S. trutta*, occurring in sympatry in the Rhone (Rho). Seven markers (in red) were detected as outliers in both comparisons across the drainage boundary but not between the two species within the Rhone. For these markers, the average band frequency in each group is given in panel **b**).

Both trout species from the secondary sympatry site in the Rhone showed a high proportion of outliers in the comparison with *S. trutta* from the Rhine (≥6.5%), but not between each other (Figure 3). Seven AFLP markers were significant outliers in both Rhone-Rhine comparisons, and only two of these showed elevated but non-significant F_ST_ values also in the comparison between the sympatric Rhone taxa (Figure 4a). The most extreme AFLP band frequencies at these seven loci were observed in *S. trutta* from the Rhine and *S. rhodanensis* from the Rhone respectively, with intermediate frequencies in *S. trutta* from the Rhone (Figure 4b). Intermediate to high outlier proportions above 2% were observed in four of the comparisons involving drainages from opposite sides of the Alps (Rhone/Rhine vs Ticino/Poschiavino), and low values ≤1.1% in three pairwise analyses involving the Ticino (Figure 3).

For outlier AFLP loci with intermediate band frequencies within drainages (0.25-0.75), we calculated pairwise linkage indices [33] to investigate potential associations between markers. Overall, this analysis was constrained by the low levels of polymorphism often observed within individual drainages at the outlier loci. Among the 52 linkage indices computed, around two thirds (67.3%) fell between 0.4 and 0.6, and the most extreme value observed was 0.2, suggesting limited linkage at least among some of these AFLP outliers.

Two outlier AFLP markers (Loc38, Loc53) were regionally fixed for alternative variants (e.g. minor variant ≤2.5%; Table 2). In both cases, the most pronounced difference was observed between the *S. rhodanesis* genotypes from the Rhone and *S. trutta* from the Rhine. The remaining markers were either polymorphic everywhere (14%) or fixed in some area(s) and polymorphic in others (86%). Overall, the average band frequency varied significantly more across the five groups for markers detected as outliers in at least one between-group comparison than for non-outliers (mean difference between maximum and minimum band frequency: outliers: 0.72; non-outliers: 0.27; Mann–Whitney U test, P<0.001).

**Table 2:** Average band frequencies per drainage and species for all markers detected as a significant outlier in at least one between-drainage comparison

**Marker ID**	**Average AFLP band frequency**					**Outlier in**
	**P**	**T**	**Rhi**	**Rho(*trutta*)**	**Rho(*rhod*)**	
**Loc18**	0.551	0.143	**0.014**	0.517	0.933	Rhi-Rho(*trutta*); Rhi-Rho(*rhod*)
**Loc29**	0.859	0.619	0.558	0.207	0.200	P-Rho(*rhod*)
**Loc33**	0.474	**0.016**	**0.007**	**0.000**	**0.000**	P-Rho(*rhod*)
**Loc37**	0.038	0.079	**0.014**	0.483	0.929	Rhi-Rho(*trutta*); P-Rho(*rhod*); Rhi-Rho(*rhod*)
**Loc38**	0.038	**0.016**	**0.014**	0.321	1.000	Rhi-Rho(*trutta*); P-Rho(*rhod*); Rhi-Rho(*rhod*); T-Rho(*rhod*)
**Loc39**	**0.000**	**0.016**	**0.000**	0.286	0.786	Rhi-Rho(*trutta*); P-Rho(*rhod*); Rhi-Rho(*rhod*)
**Loc40**	0.910	0.952	1.000	0.655	0.333	Rhi-Rho(*trutta*); Rhi-Rho(*rhod*)
**Loc41**	0.026	0.032	**0.000**	0.179	0.929	Rhi-Rho(*trutta*); P-Rho(*rhod*); Rhi-Rho(*rhod*); T-Rho(*rhod*)
**Loc42**	0.128	**0.016**	**0.007**	0.464	0.929	Rhi-Rho(*trutta*); P-Rho(*rhod*); Rhi-Rho(*rhod*); T-Rho(*rhod*)
**Loc43**	0.526	0.095	**0.007**	**0.000**	**0.000**	P-Rho(*rhod*); P-Rhi
**Loc44**	0.701	0.946	1.000	0.966	0.533	P-Rhi
**Loc45**	1.000	1.000	0.986	1.000	0.600	P-Rho(*rhod*)
**Loc45**	0.564	0.238	**0.020**	**0.000**	0.133	P-Rho(*rhod*)
**Loc46**	0.064	0.095	0.218	0.586	1.000	P-Rho(*rhod*); T-Rho(*rhod*)
**Loc47**	0.987	1.000	0.993	0.724	0.533	Rhi-Rho(*trutta*)
**Loc48**	1.000	1.000	1.000	0.862	0.267	P-Rho(*rhod*); Rhi-Rho(*rhod*); T-Rho(*rhod*)
**Loc48**	0.935	1.000	1.000	0.897	0.357	Rhi-Rho(*rhod*)
**Loc49**	0.974	0.921	0.959	0.828	0.071	P-Rho(*rhod*)
**Loc50**	0.872	1.000	1.000	0.931	0.333	Rhi-Rho(*rhod*); T-Rho(*rhod*)
**Loc50**	0.359	0.032	0.034	0.345	0.867	Rhi-Rho(*trutta*)
**Loc51**	0.895	0.968	1.000	0.966	0.357	Rhi-Rho(*rhod*)
**Loc52**	0.487	0.444	0.639	0.897	1.000	P-Rho(*rhod*)
**Loc53**	0.282	0.079	**0.014**	0.241	1.000	P-Rho(*rhod*); Rhi-Rho(*rhod*); T-Rho(*rhod*)
**Loc54**	0.128	0.190	0.265	0.517	1.000	P-Rho(*rhod*)
**Loc55**	**0.013**	0.032	**0.020**	0.207	0.867	P-Rho(*rhod*)
**Loc56**	0.436	0.127	**0.007**	0.172	0.933	Rhi-Rho(*rhod*)
**Loc57**	0.962	1.000	1.000	0.931	0.333	Rhi-Rho(*rhod*)
**Loc58**	0.513	0.095	0.027	**0.000**	**0.000**	P-Rho(*rhod*)
**Loc59**	0.141	0.194	0.422	0.679	0.929	P-Rho(*rhod*)
**Loc60**	0.192	0.254	**0.000**	**0.000**	**0.000**	T-Rhi

Bold font indicates fixed band absence (≤2.5% frequency of AFLP band) and underlined font fixed band presence (≥97.5%). P=Poschiavino; T=Ticino; Rhi=Rhine; Rho(*trutta*)=*S. trutta*-like genotypes from Rhone; Rho(*rhod*)=*S. rhodanensis*-like genotypes from Rhone.

### Evidence of (parallel) adaptive divergence along altitudinal gradients

We used two complementary approaches to investigate replicated patterns of potentially adaptive genetic differentiation along multiple altitudinal gradients. First, outlier scans in Bayescan were conducted within each drainage and, in a second step, within each of the three rivers sampled within the Rhine. By far the highest number of outliers (13) was observed within the Rhine system (Table 1), while only one outlier was detected in the Ticino and none in the Rhone and Poschiavino. The logarithm of the posterior odds (Table 1) for markers significant in one drainage was typically around or below zero in all other drainages consistent with neutrality. Separate outlier scans for the three rivers sampled within the Rhine resulted in zero to five outliers per river (six in total), which did not correspond in all cases to the markers significant at the drainage scale (Table 1).

**Figure 5:**
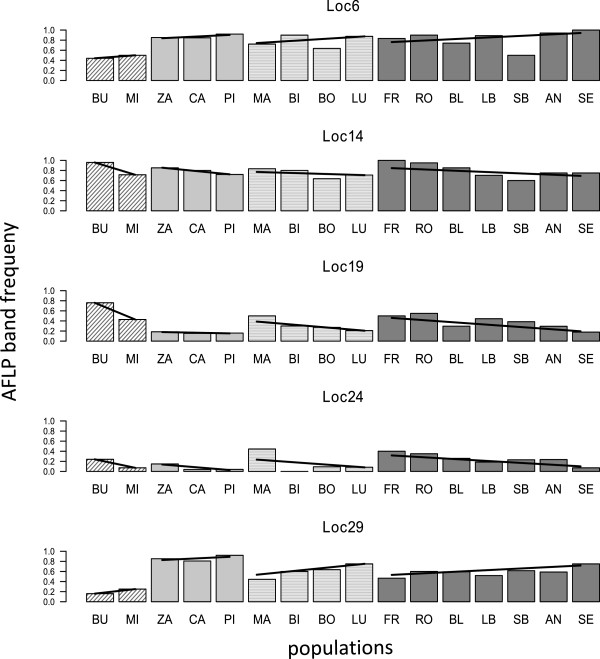
**AFLP band frequencies for the five markers showing replicated associations with altitude. **Estimated AFLP band frequencies in the 16 populations of European trout sampled along Alpine altitudinal gradients for five markers showing replicated patterns in each drainage. The shading indicates different major river systems (hatched=Rhone; light grey=Poschiavino; light grey with horizontal stripes=Ticino; dark grey=Rhine). Within drainages, sites are ordered by increasing altitude. Site identifiers correspond to Figure 1. Lines show fitted values from a logistic regression model calculated separately for each drainage.

In a second analysis, we tested each AFLP marker for associations between band frequency and altitude of the sampling site using a permutation scheme which accounted for the hierarchical structure in the data. Significant test results were obtained for 17 markers (Table 1). At five of these loci, the same pattern (i.e. increase/decrease of band frequency with altitude) was replicated in each of the four drainages (Figure 5). To test for non-independence between these five markers, the linkage index of Gaudeul et al. [33] was calculated between all pairs with intermediate band frequencies (0.25-0.75) within a given population. In total, 41 estimates were obtained which all ranged between 0.25 and 0.7 suggesting only limited association between the markers where such a test was possible.

Within the Rhine alone, we tested for associations between altitude and AFLP genotype using a logistic regression analysis and identified eight markers as significant (Table 1). Three of them also showed evidence of divergent selection in the outlier scans (Loc1, Loc12, Loc17; Table 1).

### Adaptive divergence does not increase with geographical distance or altitudinal contrast

Within the Rhine, we used a partial Mantel test to investigate if the level of adaptive divergence between populations was influenced by the altitudinal or geographical distance between sites. Adaptive differentiation was approximated by the proportion of AFLP and microsatellite outliers identified in Bayescan analyses of all 21 possible pairwise comparisons. Neither of the two explanatory variables was significantly associated with the proportion of outliers with the two variables explaining only 1.45% of the total variance.

## Discussion

### Remnants of phylogeographic structure in spite of human-mediated dispersal

Historically, each of the three major European drainage systems examined in this study would have harbored different evolutionary lineages of trout (Figure 1) which were characterized by considerable genetic divergence [21] and are considered four distinct species: historically allopatric *S. trutta*, *S. rhodanensis* and *S. marmorata*/*S. cenerinus*, where the latter two were historically sympatric within the Po drainage. Today, we still detect some genetic subdivision between major drainages most of which probably reflects differences in the relative contribution of these species to the local gene pools (Figure 2). However, the molecular data also clearly show that genotypes typical of Rhine populations of *S. trutta* occur, at variable proportions, in the two Mediterranean drainages. The frequency of *S. trutta*-like genotypes was particularly high in the Ticino, which, historically, would have contained *S. marmorata* and *S. cenerinus* just as the Poschiavino (e.g. [23,26,34]). These AFLP-based patterns are fully consistent with previous results from allozymes and microsatellite markers [27-29].

The area-wide distribution of *S. trutta*-like genotypes almost certainly reflects the directionality of historical stocking activities, which involved large-scale introductions of Atlantic *S. trutta* into the Mediterranean systems. Occasional translocations in the opposite direction appear to have taken place as suggested by the detection of Po-like genotypes in population SE in the Rhine (Figure 2). In fact, this population contains individuals with mitochondrial haplotypes typical of Danubian trout and marble trout, *S. marmorata*, one of the two trout species endemic to the Adriatic basin (our own unpublished data).

The observed geographical structure and directionality of gene flow suggests that the genetic variation now available to selection falls into two main groups: i) variants present in all major drainages and ii) variants restricted to the Rhone or the Po respectively. The first group would include ancestral variation and variants historically associated with the Atlantic *S. trutta*, and it is for these variants that patterns of parallel adaptation would be most readily detectable in our analyses.

### Evidence of adaptive divergence between drainage systems

The extent and nature of environmental differences between drainages is not well understood. However, it seems likely that such differences do exist and that the extended periods of allopatric divergence between some of the trout species before human-mediated secondary contact and admixture would have facilitated the evolution of traits advantageous in a particular drainage. Loci experiencing such long-term divergent selection would eventually become regionally fixed for alternative alleles producing a pattern where a given genetic variant is specific to a particular trout species. Only two of our markers show such fixed differences between regions (Table 2).

Under an alternative and perhaps more likely scenario individual loci would experience directional selection in some environments (i.e. some drainage systems) but no selection in others. If adaptation was often from standing genetic variation, this could lead to the fixation of the favoured variant in one region and the, at least transient, retention of ancestral variation in the other. Consistent with such a scenario, 86% of the markers detected as outliers in at least one between-drainage comparison, were fixed for a particular variant in some region(s) and polymorphic elsewhere (typically the Rhone and/or Po; Table 2). It is currently not possible to determine if, at these loci, ancestral variation has been retained in the Mediterranean drainages but not in the Rhine system or if novel variation has been introduced into the Mediterranean systems by recent unidirectional gene flow as a result of human management practices.

**Figure 6:**
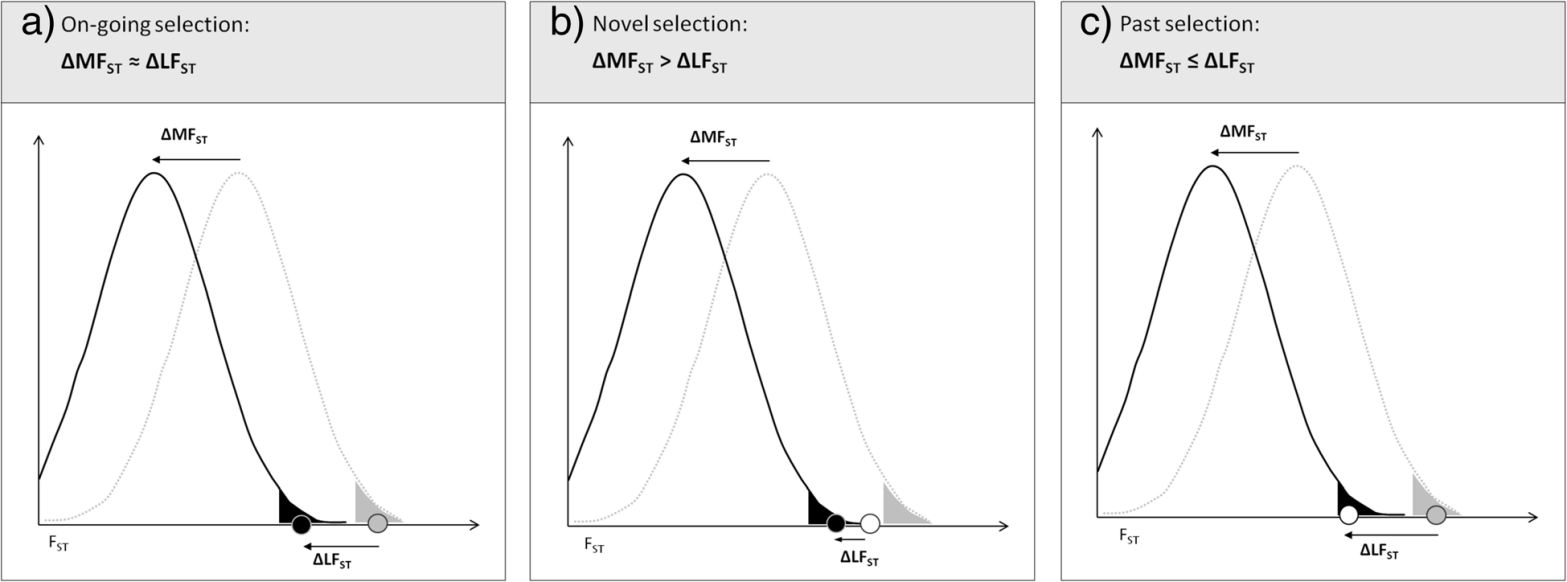
**Potential scenarios for genetic patterns of differentiation detected after admixture between two diverged lineages. **F_ST_ is calculated between two populations before (grey) and after admixture (black) for a large number of loci. As a result of the admixture, the current mean differentiation is reduced compared to the historical mean by an amount ΔMF_ST_. The F_ST_ observed at individual loci will also change but the difference between the current and the historical value (ΔLF_ST_) will depend on the selection regimes before and after admixture. Note that filled circles indicate loci under divergent selection, and empty circles indicate loci evolving neutrally at a given point in time. In a genome scan carried out today (i.e. after admixture), a locus would fall within the tail of the current F_ST_ distribution (black shaded area) and be considered an outlier under one of the following scenarios: **a) On-going selection** before and after admixture. **b) Novel selection** only after admixture. **c) Past selection** only before admixture, representing the ‘ghost of selection past’. Under scenario “c”, the outlier status of the locus will be transient.

Still, it seems likely that a locus showing elevated between-population differentiation today compared to the genome-wide average has experienced divergent selection at least during some of its evolutionary history. However, in cases like the trout where diverged lineages came into secondary contact, it may be difficult to distinguish between historical and contemporary effects of selection, and hence the role of past versus current environments, based on the results of an outlier scan alone. On the one hand, the current outlier status of a marker in a zone of secondary admixture between historically divergent species may be indicative of contemporary divergent selection on this locus which may (but need not) have been selectively neutral in the past (Figure 6a,b). Alternatively, markers fixed or nearly fixed for alternative variants in different species - as a result of divergent selection in the past - may transiently retain elevated levels of differentiation after secondary contact and genetic admixture, even if selectively neutral today, representing the ‘*ghost of selection past*’ (as illustrated in Figure 6c; see also [35]).

The history of unidirectional stocking and the associated current coexistence in secondary contact of two distinct genetic clusters within the Rhone allow us to test if, at some loci, different genetic variants are favoured in the two drainages. In that case, we would predict that a locus should be detected as an outlier in both Rhone vs Rhine comparisons. Within the Rhone, however, the locally advantageous *S. rhodanensis* variant should be favoured also in individuals with an otherwise *S. trutta*-like genotype and, consequently, the marker would show low genetic differentiation between the two Rhone groups. Evidence for such a pattern is provided by five markers which were outliers in both comparisons across the drainage boundary but showed F_ST_ values around or below the average in the comparison between the two species in sympatry in the Rhone (Figure 4a). All of these markers are fixed or nearly fixed for alternative variants in the *S. rhodanensis*-like cluster from the Rhone and *S. trutta* from the Rhine while the *S. trutta*-like cluster from the Rhone is intermediate (Figure 4b), consistent with a pattern of adaptive introgression from *S. rhodanensis* into *S. trutta* within the Rhone.

### Adaptive divergence and the potential for gene flow between populations

At a regional scale within the Rhine drainage, we found that between 0 and 4% of the examined loci showed evidence of divergent selection between population pairs within and between rivers. However, this proportion was independent of both the potential for gene flow (approximated by pairwise waterway distances) and the altitudinal difference between sites. In contrast, many other studies have suggested that the homogenising effects of gene flow may often constrain adaptive divergence between natural populations (reviewed e.g. in [4,36]). We see possible explanations for our different result.

First, our analysis implicitly assumes that the intensity of divergent selection between altitudes is relatively homogeneous across space. This assumption may be violated in some cases. In fact, the outlier behaviour of several markers appears to be driven by one (or a few) populations that differ markedly in average AFLP band frequency from all other populations. Similar patterns were observed at some microsatellite loci in an earlier analysis of a dataset from a larger number of populations in the same area (see e.g. Figure 2b of [29]), and they may point to differences in local selection regimes that are not consistently related to altitude, or to differences in the genetic mechanism of adaptation to the same local selection regimes. It is also likely that a certain proportion of the outliers detected in our analysis are false positives. In fact, a common approach to confirm the results of outlier scans is to look for replicated patterns in independent comparisons across similar ecological gradients (see below and e.g. [18]) which, of course, is not possible in the case of local effects.

Secondly, the rates of gene flow between the different Rhine populations may have been too homogeneous to detect an effect on adaptive divergence. The proportion of outliers is certainly a crude measure of adaptive divergence and a wider range of parameter values may need to be investigated to detect significant patterns. Indeed, a recent study at a larger geographical scale in Denmark (maximum distance between sites ca. 1000km compared to ca. 450km in this study) did find a positive correlation between the number of outliers and the geographical distance between populations [32]. Still, the proportion of outliers detected in a genome scan may be a useful measure of the extent of adaptive population divergence in many natural systems where it is not possible to perform reciprocal transplant experiments.

We find that the proportion of outliers increases when we compare populations (species) from different major drainages, which were fully isolated for extended periods in their evolutionary history. This is illustrated by the on average, higher proportion of outliers in the between-drainage comparisons (Figure 3) than along gradients within rivers (Table 1), although these results may not be strictly comparable due to differences in statistical power. However, the pattern holds also in comparisons between pairs of populations. Specifically, the average proportion of outliers between six randomly selected population pairs from different drainages was double that observed among the intraspecific *S. trutta* comparisons made within the Rhine (2.8% vs 1.4%). These comparisons do not allow us to distinguish between the effects of variation in geographical isolation and the effects of variation in time available for divergence, both of which are generally much larger between these drainages than within. Finally, the strength of divergent selection could be larger between drainages than within.

### Evidence of parallel adaptive divergence along altitudinal gradients

One of the strongest types of evidence for the action of natural selection is provided by patterns of parallel evolution (e.g. [37]). Therefore, we concentrate our search for evidence of adaptive divergence along altitudinal gradients on trends in genotype-altitude associations that are paralleled across drainage systems. The most convincing support for such parallel patterns stems from the five markers where the frequency of the AFLP band showed consistent trends with altitude in all four drainages (Figure 5). Our analysis provided no evidence of linkage between these five markers. Additional evidence for parallel altitudinal patterns of adaptive divergence comes from several outliers within the Rhine drainage, which also show significant associations between AFLP band frequency and altitude (Table 1). Finally, our previous analyses based on microsatellite markers detected replicated altitudinal changes for two alleles at a locus near the MHC I region [29].

Together, these results suggest that altitude may well be an important source of divergent selection within single rivers, and potentially acts on multiple independent loci. If we consider only the five markers showing consistent trends in all drainages (Figure 5), we obtain an estimate of ca. 2% of the markers responding to altitude. This value is identical to the proportion of markers identified as candidate loci underlying altitudinal adaptation in the frog *Rana temporaria*[18]. It is also in line with the proportion of loci exhibiting evidence of selection in some classical examples of divergent adaptation with gene flow, e.g. between sympatric sister species of African cichlids (2–6 %; Bezault et al., *submitted*), host races of walking stick insects (1-2%; [38]) or periwinkle ecotypes (5%; [39]). In trout, with high rates of both natural and human-mediated dispersal, such divergent adaptation within species may exist in the face of extensive gene flow.

## Conclusions

Together, our results confirm that adaptive genetic divergence is common in Alpine trout. The proportion of outlier loci was particularly high between drainages and associated species, suggesting that adaptive divergence may be facilitated by strong divergent selection, geographical isolation and/or time. Still, parallel altitudinal changes of AFLP band frequencies within multiple drainages indicate that altitude may well be an important source of divergent selection within rivers and suggest divergent adaptation in the face of gene flow. In general, the amount of gene flow against which adaptive divergence can be maintained varies by orders of magnitude between different study systems. In the future it will be important to investigate how much of this variation is due to variation in the intensity or dimensionality of selection and the genetic architecture of adaptation [40]. Alpine trout could become an aquatic model system for investigating the relative importance of these constraints in adaptation to climate gradients.

## Methods

### Sampling

Trouts (*Salmo trutta* species complex) were caught by electrofishing within river sections of typically 100-200m at 16 sites (Figure 1) in Switzerland between October 2007 and December 2008. Sampling sites were arranged along replicated altitudinal transects in three major European drainages (Rhone, Rhine and Po). A single gradient was studied in the Rhone (river Allaine), three gradients in the Rhine (Reuss, Thur, Alpine Rhine) and two in the Po drainage (Ticino, Poschiavino). The investigated altitudinal range was 381-486m above sea level (asl) in the Rhone, 398-1776m asl in the Rhine, and 277-1802m asl in the Po. Besides the altitudinal difference, gradients varied in steepness. Note that the locations included in this study are a subset of those in [29] where altitudinal information and coordinates are provided for all sites.

Tissue samples were taken from a fin and stored in absolute ethanol. We specifically targeted large fish to avoid sampling recently introduced hatchery fish. The total sample size was 402 individuals with 14–30 individuals per site. All electrofishing and sampling was authorized by the responsible local fisheries authorities.

### Molecular analysis

DNA was extracted from the fin clips using a BioSprint 96 DNA Blood Kit and BioSprint 96 workstation (QIAGEN GmbH, Hilden, Germany), following the instructions of the manufacturer. The purity and concentration of the extracted DNA was quantified using a Nanodrop ND-1000 spectrophotometer (Thermo Fisher Scientific, Waltham, MA, USA). AFLP profiles were generated using the restriction enzymes EcoRI and MseI with a protocol adapted from [41] and [42]. To reduce potential bias due to plate-specific effects, individuals from each population were distributed across the different sample-plates (96-well plates), including also samples repeated independently across plates as control and to estimate reproducibility (see below). The restriction and ligation reaction was conducted in a single step. In a total volume of 11μl, 300ng DNA were combined with 5 U EcoRI, 1 U MseI, 120 cohesive end U T4 ligase, T4 ligase buffer at 1x concentration, 0.55 μg BSA (all New England Biolabs Inc., Ipswich MA, USA), 50 mM NaCl, 50 μM MseI-Adaptor, and 5 μM EcoRI-Adaptor. The adaptors were heated for 5 min at 95°C before use. The mixture was then incubated for 2h at 37°C using a Techne TC-412 PCR machine.

The pre-selective amplification was conducted using 3μl of restriction-ligation product, 0.5 μM of each primer (5’-GACTGCGTACCAATTCA-3’ and 5’-GATGAGTCCTGAGTAAC-3’), 0.4 μg BSA, 200 μM dNTP, 2.5 mM MgCl_2_, 0.75 U Taq DNA polymerase and associated buffer at 1x concentration (Bioline Ltd., London, UK). The cycling protocol consisted of an initial step at 72°C for 2 min then 20 cycles of 94°C for 20 sec, 56°C for 30 sec, and 72°C for 2 min and a final extension at 60°C for 30 min.

The selective amplification was performed in four multiplex reactions, each containing three selective primer-pair combinations. Each multiplex contained the same three EcoRI primers each labeled with a different fluorescent dye and extended with three selective bases (Primer 1: 0.07 μM, selective bases = ACA, dye = Alexa AX647; primer 2: 0.09 μM, AAG, IRD IR700; primer 3: 0.04 μM, AGC, Alexa AX750). In each multiplex, these three EcoRI primers were combined with 0.22 μM of a single MseI primer (multiplex 1: selective bases CTC; multiplex 2: CTA; multiplex 3: CAG; multiplex 4: CGC), 5 μl QIAGEN multiplex mastermix and 1.5 μl of a 1:10 dilution of the pre-amplification product in a final volume of 11.25 μl. The mix was run on a Techne TC-412 thermocycler using the following protocol: initial denaturation at 95°C for 15 min, then 10 cycles of 94°C for 30 sec, 65°C for 1 min (with the annealing temperature reduced by 1°C after each cycle), 72°C for 1 min, followed by 25 cycles of 94°C for 30 sec, 55°C for 30 sec and 72°C for 1 min, and a final extension step of 72°C for 10 min. The amplification products were run on a Beckman Coulter CEQ 8000 (Beckman Coulter Inc., Brea CA, USA) automatic sequencer together with a size standard 400 (Beckman Coulter).

The sequencer electropherograms were analysed for each multiplex separately in GeneMarker V1.85 (SoftGenetics, State College PA, USA). Bins were pre-defined automatically by the software, followed by manual adjustment based on the visual inspection of peak positions in an overlay of all traces and the reproducibility of genotypes between replicates. Fragments between 60 and 400 bp were considered. To estimate reproducibility, 12 samples were replicated within sample-plates and 28 samples between sample-plates resulting in a total of 40 replicates (10% of the total number of samples). For these replicates, the molecular protocol was carried out independently starting from the restriction-ligation reaction. Peaks were called automatically in GeneMarker, and marker-bins were deleted if mismatches in scored genotypes were observed in more than 10% of the replicates, resulting in a total of 570 reproducible markers.

Following [43], individuals with more than 15% and markers with more than 10% missing data were removed from the analysis. Markers were considered polymorphic and retained for analysis if the minor variant occurred at a frequency of ≥2.5% across all individuals. This resulted in a final dataset of 229 polymorphic AFLP markers typed in 369 individuals. The error rate across all polymorphic markers was estimated at 4.7% based on the 40 replicates.

Microsatellite genotypes were available for all individuals from a previous study [29]. The microsatellite panel included eight presumably neutral markers from different linkage groups, two markers with diagnostic allele size ranges in three of the trout lineages expected in our study area (*S. trutta*, *S. rhodanensis* and *S. marmorata*; [44]), and eight candidate markers linked to loci with a role in immune defense, thermal tolerance and spawning time in other salmonids. Additional details about the markers and the molecular protocols are provided in [29].

### Statistical analysis

#### Population structure

Structure v.2.3.1 [45,46] was used to assess the genetic population structure. We assumed an admixture model and correlated allele frequencies with 10’000 burn-in steps and 100’000 MCMC (Markov chain Monte Carlo) steps. The number of clusters was varied between K = 1–6, with the largest value corresponding to the number of sampled rivers, and five independent runs were performed for each K. The optimal number of clusters was assessed based on the probability of the data given K estimated by Structure and visual inspection of the Structure barplots. The results were averaged across replicates using Clumpp 1.1.2 [47] and graphically displayed with Distruct 1.1 [48].

An analysis of molecular variance (AMOVA) was used to quantify the proportion of genetic variance within and between different groups. We assessed two different hierarchical structures where i) populations were divided into three groups corresponding to major drainages (i.e. Rhone, Rhine, Po) or ii) into four groups with an additional split within the Po between the Ticino and Poschiavino rivers which are separated by a large waterway distance. The AMOVA was computed in Arlequin v. 3.5 [49], and the significance tests were based on 16’000 permutations.

#### Testing for divergent selection between drainages

We used a Bayesian method implemented in Bayescan v. 2.1 [50] to identify markers showing unusual levels of genetic differentiation compared to the genomic average. Outlier scans were performed for all possible pairwise comparisons between drainages and species, with populations within drainages pooled. The two geographically distant regions sampled within the Po - Ticino and Poschiavino - were treated as separate drainages here and in all subsequent analyses where hierarchical structure was considered. Population SE was excluded from the Rhine dataset because of the presence of non-Atlantic genotypes (Figure 2). Within the Rhone, the Structure analysis identified two distinct genetic clusters. One cluster was unique to the Rhone and most likely corresponds to the native *S. rhodanensis*, the other represents introduced *S. trutta* (Figure 2). These two genetic groups were treated separately in the between-drainage outlier scans. An individual was considered as *S. trutta*-like or *S. rhodanesis*-like if its estimated ancestry in one of the two clusters was at least 60%. Note that the two genetic groups show some spatial separation within the Allaine river. The *S. rhodanesis*-like group is composed almost exclusively of individuals from the downstream site BU, while the *S. trutta*-like group is dominated by individuals from the upstream site MI (see Figure 1 for location of the sites).

All analyses were run assuming even prior odds, and default settings for all other parameters. In particular, a uniform prior from 0 to 1 was used for F_IS_ as samples from multiple sites within each drainage were pooled in these analyses. A locus was considered to be an outlier if the posterior odds favouring a model with selection were above a threshold set by the software to ensure a false discovery rate of ≤20%. Identical analyses were performed on the microsatellite data.

We investigated potential linkage disequilibria between AFLP outliers within drainages in all comparisons where at least five outlier loci had been detected (five out of ten between-drainage analyses). Specifically, we calculated the linkage index of Gaudeul et al. [33] as $\frac{1}{n}\sum_{i}\left|m_{ki}–m_{ki}\right|$ where *m*_*ki*_ and *m*_*li*_ are the genotypes of individual *i* at locus *k* and *l* respectively, for all 52 pairs of outlier loci with intermediate AFLP band frequencies (i.e. 0.25-0.75) in a given drainage.

To investigate potential associations between particular AFLP variants and evolutionary trout lineages, average AFLP band frequencies were calculated for each marker in each of the five groups (Rhine, Poschiavino, Ticino, Rhone/*S. trutta*-like genotype, Rhone/*S. rhodanensis*-like genotype).

#### Testing for divergent selection along replicated altitudinal gradients

Separate outlier scans were conducted for the Rhone, Rhine, Ticino and Poschiavino rivers as well as for the three rivers sampled within the Rhine drainage (Reuss, Thur, Alpine Rhine). We did not perform a global analysis of all 16 populations as the hierarchical population structure present in the data could lead to a strong increase in the number of false positives [51]. Bayescan was run assuming even prior odds and an upper and lower bound on F_IS_ of 0 and 0.028 respectively. These values correspond to the limits of the 99% confidence interval for F_IS_ estimated from microsatellite markers. We used the same thresholds as above to identify markers showing evidence of selection. In the Ticino, two nearby samples with low sample size (BO, BI) were pooled for the analysis.

Associations between band frequency at a given AFLP marker and altitude of the sampling site were further investigated with a permutation test implemented in R [52]. This test asked how frequently one would detect by chance a pattern of consistent decrease/increase in band frequency between consecutive sites that is at least as extreme as our observation given the band frequencies within populations and given the hierarchical structure in the data. Specifically, sites were ordered by altitude within drainages and band frequencies were compared between consecutive sites within drainages. A population pair was assigned a value of +1 if the band frequency increased by more than a threshold value of 0.01, -1 if it decreased by more than 0.01, and 0 otherwise. These values were summed to obtain a measure of association between band frequency and altitude. The observed value was then compared to a distribution obtained by randomising the order of sites within drainages and recomputing the measure of association as described above. A total of 5’000 permutations were performed for each marker, and we assessed the proportion of permutations giving a value for the summary statistic which was more extreme than the observed (corresponding to a one-tailed test). Note that, in principle, this question could be addressed with a logistic analysis of covariance with altitude and drainage as explanatory variables. We decided against this approach because of the small number of sampling sites in some of the drainages.

A separate logistic regression analysis was performed for the Rhine populations. We found that this analysis produced a large number of significant results (44 out of 148 tests with P<0.05) while our permutation test was much more conservative (7 out of 229 tests with two-sided P<0.05). Because of this difference between the two methods, we use an ad-hoc approach with different significance thresholds for the two tests (permutation test: 5% one-tailed without multiple test correction; drainage-specific logistic regression: 5% with Bonferroni correction).

#### Factors promoting adaptive divergence

We used all 21 possible pairwise comparisons between our sampling sites in the Rhine to investigate to what extent adaptive divergence between populations was promoted by environmental contrasts and/or spatial isolation. As a measure of adaptive divergence, we used the proportion of outliers between a given population pair. Analogous outlier scans were conducted for the microsatellite markers, and the results from the two analyses were combined. The absolute difference in altitude between two sites (in m) was used as a measure of environmental contrast, and the waterway distance (in km) as a measure of dispersal opportunity. The association between the proportion of outliers and the two explanatory variables was investigated using a partial Mantel test in Fstat v.2.9.3.2 [53].

## Authors’ contributions

IK was involved in the design of the study, the collection of samples and molecular data, performed some of the statistical analyses and drafted the manuscript. JS participated in the field work, performed the molecular and some of the statistical analyses and helped to draft the manuscript. EB contributed to the collection of the molecular data, their statistical analysis and the drafting of the manuscript. OS conceived of the study, participated in its design, provided advice on data analysis and helped to draft the manuscript. All authors read and approved the final manuscript.
